# Investigation of the Solid-State Interactions in Lyophilized Human G-CSF Using Hydrogen–Deuterium Exchange Mass Spectrometry

**DOI:** 10.1021/acs.molpharmaceut.3c01211

**Published:** 2024-03-22

**Authors:** Victoria E. Wood, Mark-Adam Kellerman, Kate Groves, Milena Quaglia, Elizabeth M. Topp, Paul Matejtschuk, Paul A. Dalby

**Affiliations:** † Department of Biochemical Engineering, University College London, London WC1E 6BT, United Kingdom; ‡ Standardisation Science, NIBSC, Medicines & Healthcare Products Regulatory Agency, South Mimms, Hertfordshire EN6 3QG, United Kingdom; § LGC, Queens Road, Teddington, Middlesex TQ11 0LY, United Kingdom; ∥ Department of Industrial and Molecular Pharmaceutics, College of Pharmacy, and Davidson School of Chemical Engineering, College of Engineering Purdue University, West Lafayette, Indiana 47907, United States

**Keywords:** solid-state hydrogen−deuterium exchange mass spectrometry (ssHDX-MS), excipient selection, sucrose, mannitol, phenylalanine

## Abstract

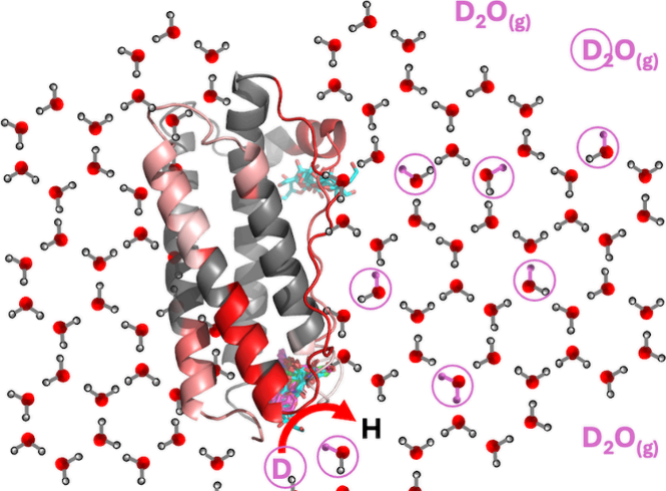

Hydrogen/deuterium exchange mass spectrometry (HDX-MS) previously elucidated the interactions between excipients and proteins for liquid granulocyte colony stimulating factor (G-CSF) formulations, confirming predictions made using computational structure docking. More recently, solid-state HDX mass spectrometry (ssHDX-MS) was developed for proteins in the lyophilized state. Deuterium uptake in ssHDX-MS has been shown for various proteins, including monoclonal antibodies, to be highly correlated with storage stability, as measured by protein aggregation and chemical degradation. As G-CSF is known to lose activity through aggregation upon lyophilization, we applied the ssHDX-MS method with peptide mapping to four different lyophilized formulations of G-CSF to compare the impact of three excipients on local structure and exchange dynamics. HDX at 22 °C was confirmed to correlate well with the monomer content remaining after lyophilization and storage at −20 °C, with sucrose providing the greatest protection, and then phenylalanine, mannitol, and no excipient leading to progressively less protection. Storage at 45 °C led to little difference in final monomer content among the formulations, and so there was no discernible relationship with total deuterium uptake on ssHDX. Incubation at 45 °C may have led to a structural conformation and/or aggregation mechanism no longer probed by HDX at 22 °C. Such a conformational change was observed previously at 37 °C for liquid-formulated G-CSF using NMR. Peptide mapping revealed that tolerance to lyophilization and −20 °C storage was linked to increased stability in the small helix, loop AB, helix C, and loop CD. LC-MS HDX and NMR had previously linked loop AB and loop CD to the formation of a native-like state (N*) prior to aggregation in liquid formulations, suggesting a similar structural basis for G-CSF aggregation in the liquid and solid states.

## Introduction

Protein dynamics can be studied using hydrogen–deuterium exchange as measured by mass spectrometry or NMR (HDX-MS or HDX-NMR). First demonstrated by Linderstro̷m-Lang,^1^ HDX-MS can report on both global and local dynamics in proteins. Compared to NMR, HDX-MS brings higher sensitivity at low protein concentrations, no limitations due to protein size, and detection of multiple coexisting protein conformers.^2^ HDX primarily reports on amide hydrogen exchange with solvent deuterons, catalyzed by acid, base, or water. Backbone amide hydrogens buried in the protein interior or forming highly stable hydrogen bonds exchange slowly compared with surface amide hydrogens or those involved in weak hydrogen bonds. Amide hydrogen exchange, therefore, provides information on protein flexibility, conformational distributions, hydrogen-bond patterns, and structure.^3^

More recently, solid-state hydrogen–deuterium exchange coupled with mass spectrometry (ssHDX-MS) has enabled a detailed analysis of protein structure and matrix interactions within amorphous solid powders produced for example by lyophilization,^4^ or spray drying.^5^ For solid-state exchange, vials containing a lyophilized protein formulation are placed uncapped in a sealed desiccator over a saturated salt solution of D_2_O to maintain constant D_2_O in the vapor phase. Samples are then removed at various times and stored at −70 °C, prior to reconstitution under quench conditions and analysis of either the intact or pepsin-digested protein by mass spectrometry. The global rate of exchange, measured immediately after lyophilization, has been shown to correlate with protein aggregation and chemical degradation during storage for up to 1 year with formulated model proteins such as myoglobin^6^ and for over 2 years with therapeutically relevant monoclonal antibodies.^7,8^ The ssHDX-MS method has also been able to identify differences between mAbs formulated by spray drying and three different lyophilization processes, demonstrating its potential for monitoring and quality control in these processes.^9^

Backbone amide uptake of deuterium for protein in lyophilized solids is likely to differ from that in solution for several reasons. D_2_O sorption and diffusion processes result in slower exchange during HDX-MS labeling within amorphous solids than that in aqueous solutions.^10^ However, previous ssHDX showed completion of moisture sorption within a few hours for mAb formulations with sucrose and/or mannitol and that this did not contribute significantly to exchange kinetics beyond this time, suggesting that the rate and extent of exchange were not simply dependent on D_2_O adsorption.^11,12^

The kinetics of deuterium incorporation during ssHDX-MS are likely to report on the wider hydrogen bond network linking the protein to the amorphous solid state, which includes both intramolecular hydrogen bonds for the native protein and intermolecular hydrogen bonds to the surrounding matrix.^4^ Water replacement is thought to occur in the amorphous solid state, whereby proteins become stabilized by the formation of hydrogen bonds to excipients,^13^ and so deuterium can reach the protein either by local interactions between sorbed D_2_O and protein amides or by conduction of deuterons through hydrogen-bond networks within the solid. The extent of hydrogen bonding between the protein and excipients can depend on the excipient structure, molecular weight, and physical morphology of the solid. Residual moisture in the amorphous solids is also important as this can form hydrogen bonds with the protein and also lower the *T*_g_. The stability of proteins in amorphous solids is thought to be influenced by hydrogen bonds between protein and excipient and also between protein and water.

The rates for amide opening events and exchange observed by HDX-MS may be altered in the solid state, compared to that in solution, due to fewer dynamic modes being available. However, results to date suggest that for ssHDX, amide hydrogen atoms that do not participate in hydrogen bonds or that form relatively weak hydrogen bonds to water or excipient can exchange rapidly. Slow exchange is thought to result from amide hydrogen atoms within stronger hydrogen bonds within the protein or to excipients, with zero exchange for the strongest structural hydrogen bonds buried within the protein core.^4^

G-CSF is a widely used protein therapeutic, used to stimulate white cell proliferation after chemotherapy.^14^ However, the bacterially expressed form is sensitive to lyophilization-induced stresses^15^ and thus provides an ideal model for studying formulation methods and the impact of excipient addition and lyophilization on protein stability. A previous screen of G-CSF with various excipients, buffers, and pH, using ultrascale-down (USD) lyophilization methods, generated a wide range of formulations with different levels of survival through the lyophilization process as determined by bioactivity and monomer retention.^16^ HDX-MS has provided mechanistic insights into the stabilization of liquid G-CSF formulations and identified areas for stability re-engineering.^17^ We also recently developed an LC-MS HDX protocol for liquid-formulated G-CSF with high sequence coverage and mapped the local binding sites for several excipients.^18^ Kinetic models fitted to experimental aggregation of G-CSF in liquid formulations suggested that partial unfolding to an intermediate or native-like state (N*) was rate limiting.^19^ Peptide mapping HDX-MS for a wide range of G-CSF variants also revealed structural changes in loop AB and loop CD consistent with an N* state on the aggregation pathway, and which partially revealed a region of helix B and the beginning of loop BC.^20^ Here, we build upon this work and use ssHDX-MS to identify G-CSF-excipient interactions and local structural changes within lyophilized formulations and relate them to the stability during storage in the solid state. This work highlights similarities between structural changes linked to aggregation in the liquid and solid states and identifies potential mechanistic routes through which excipients stabilize proteins in the lyophilized formulations. This deeper understanding could potentially lead to more predictive formulation design approaches *in silico*.

## Materials and Methods

Chemicals were obtained from the following manufacturers: citric acid, phenylalanine, sodium citrate, sucrose, deuterium oxide (D_2_O) 99.9% (Sigma-Aldrich Co., Gillingham, UK), mannitol, NaCl (Fisher Scientific Inc., Loughborough, UK), and TCEP (Thermo Fisher, Hemel Hempstead, UK).

### Expression and Purification of G-CSF

G-CSF (accession code M17706) was expressed as inclusion bodies in *Escherichia coli* BL21 (DE3) cells (New England Laboratories, Massachusetts, USA) harboring a modified pET21A plasmid (Novagen, Wisconsin, USA), and confirmed by intact mass spectrometry (LC-MS), exactly as described previously.^18^ Briefly, cells were grown in 400 mL of Terrific Broth and 1 mM ampicillin, at 37 °C, within 2 L baffled shake flasks, and induced with 1 mM IPTG at OD_600_ = 0.6. After 3.5 h, cells were pelleted at 5410*g* for 30 min at 4 °C (Avanti J-20XPI; Beckman Coulter Inc., Fullerton, California, USA) and then washed, refolded, purified by size exclusion chromatography, and concentrated to 1.0 mg/mL in 10 mM sodium acetate, pH 4.25 as described previously.^18^ For each new formulation, samples were buffer exchanged into 50 mM citrate pH 4.25 with 10 kDa cutoff Slide-A-Lyzer Dialysis cassettes (Fisher Scientific, Leicestershire), mixing 1:1 with sterile-filtered 2× excipient solutions in 50 mM citrate pH 4.25 to obtain final formulated 0.3 mg/mL G-CSF, and incubated on the bench at RT (22 °C) for 1 h to ensure full equilibration. Monomer, dimer, and aggregate content was measured using analytical SEC and LC-MS for all G-CSF samples.

### Lyophilization

G-CSF formulations were filled to 200 μL in 2 mL glass vials (Schott VC002) with 13 mm-diameter igloo halobutyl-rubber stoppers (West Pharma, purchased with vials from Adelphi Pharmaceutical Packaging, Haywards Heath, UK) and placed on the lyophilizer shelf of a VirTis Genesis 25EL lyophilizer (Biopharma Process Systems, Winchester, UK) along with equivalent control formulations having no G-CSF. Thermocouples in the control vials measured sample temperatures during lyophilization. Samples were lyophilized according to the 2-day cycle in Table 1. Vials were then backfilled with nitrogen and stoppered. Samples were then subjected to ssHDX immediately or otherwise stored at −70, −20, and 45 °C for the 0 s labeling (no exchange) and 30-day storage stability studies.

**Table 1:** 42 h Lyophilization Cycle Using a VirTis Genesis Lyophilizer and 2 mL Vials Filled to 200 μL

**stage**	**step**	**temp (°C)**	**time (min)**	**vac (mTorr)**	**ramp/hold**
freeze	1	20	30		H
	2	–45	120		R
	3	–45	240		H
primary drying	1	–45	30	150	H
	2	–45	30	70	H
	3	–25	60	70	R
	4	–25	1200	70	H
secondary drying	1	30	480	70	R
	2	30	420	20	H

### Solid-State HDX

Vials were opened and placed at the edges of 2.4 L borosilicate glass DURAN desiccators, prepared the day before with 70 mL of 99.9% D_2_O and potassium carbonate (VWR Chemicals, Leicester, UK) in excess for a water activity (*a*_w_) of 0.43 (43% RH). Triplicate samples were incubated at 22 °C for each time point spanning 30 to 240 min and then immediately stoppered, flash frozen in liquid nitrogen, and stored at −70 °C. For the 0 s labeling time point, vials were immediately stored at −70 °C after lyophilization.

### Reconstitution and LC-MS of ssHDX Samples

After −70 °C storage, the samples were placed on dry ice. For each LC-MS analysis, a sample was defrosted by hand, opened, reconstituted with 2 mL of ice-cold 0.2% (v/v) formic acid, and vortexed for 10 s. Next, 50 μL was added to 50 μL of ice-cold quench solution (50 μL of 4 M guanidine hydrochloride, 600 mM tris(2-carboxyethyl)phosphine (TCEP), in 100 mM sodium acetate, pH 2.5, at 4 °C) and mixed within a high-recovery HPLC vial and loaded onto a refrigerated (0 °C) nanoACQUITY UPLC with HDX technology (Waters, Milford, Massachusetts, USA) for injection into the sample loop of the LC-MS system, with no further sample preparation or precleaning of the syringe to reduce the time from vial to injection. Blanks of 0.2% (v/v) formic acid were injected between sets of samples. A 5 μm, 2.1 mm × 30 mm Enzymate BEH pepsin column (Waters, Milford, Massachusetts, USA) at 25 °C was placed in-line between the injection valve and the trap valve to perform digestion. Resulting peptides were eluted with 0.05% formic acid at 80 μL min^–1^ into a reverse-phase VanGuard pre-column (Waters, Manchester, UK) and then eluted into an ACQUITY UPLC BEH C18 (1.0 mm × 100 mm, 1.7 μm particle diameter (Waters, Manchester, UK), at 0 °C. Peptides were resolved using a linear gradient from 8% ACN, 0.1% FA, to 35% over 7 min at 100 μL min^–1^. The eluent was directed into a Synapt G2Si ESI-Q-TOF-MS mass spectrometer (Waters, Milford, Massachusetts, USA) with electrospray ionization and postacquisition lock mass-corrected using the 2+ charge state of [Glu1]-fibrinopeptide B infused at 100 fmol/μL, 90°, to the analytical sprayer.^21^

### Data Analysis

ProteinLynx Global Server (PLGS) software v3.02 (Waters, Milford, Massachusetts, USA) generated peak lists from MS^E^ data, allowing for the oxidation of methionine. DynamX v3.0 (Waters, Milford, Massachusetts, USA) generated the HDX-MS peptide maps, including only those peptides observed in at least four of five injection repeats. Peptides were identified using the WT peptide map from MS files imported into DynamX 3.0, and the stacked spectral plots were analyzed together.

Deuterium exchange was analyzed using DynamX but validated and curated manually as described previously.^22^ No corrections were made for back exchange, as all comparisons were made relative to a G-CSF control formulation.

The differential in relative uptake between control and excipient-containing formulations was calculated as
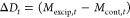
1where *M*_excip,*t*_ and *M*_cont,*t*_ are the mean of triplicate measurements for uptake at time *t* in excipient-containing and control samples, respectively.

Residue-level differential uptake, Δ*D*_res_, was estimated from weighted contributions of all peptides containing the residue:
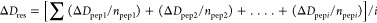
2where Δ*D*_pep*i*_ is the differential uptake for peptide *i*, *n*_pep*i*_ is the length minus 1 of peptide *i*, and *i* is the total number of peptides containing the residue.

### Freeze-Drying Microscopy (FDM) Measurements

The thermal collapse (*T*_c_) of freeze-dried material was measured by FDM using an FDCS 196 stage (Linkam, Surrey, UK), a BX51 Olympus optical microscope with TMS 94, VC 94, and liquid nitrogen pumping (LNP) control units. Samples were placed in a quartz glass crucible with a metal shim under a 13 mm glass coverslip and mounted into the sample holder for imaging with a 20× lens. Samples were frozen to −50 °C at a ramp rate of 10 °C/min, held for 5 min at −50 °C, and then at 0.1 mbar for 5 min at −50 °C, and finally the temperature ramped to 25 °C at 5 °C/min. Images were taken every 5 s during freezing and every 2 s during drying to identify the collapse point and associated *T*_c_.

### Modulated Differential Scanning Calorimetry (DSC)

For modulated DSC, triplicate samples were added to individual 80 μL steel hermetic pans with lid and O-ring and crimped. Pans were weighed before and after sample addition. DSC was performed on a Q2000 DSC (TA Instruments, Wilmslow, UK) using an empty crimped pan as a reference. An isothermal hold for 2 min was followed by cooling to −90 °C at 10 °C/min, modulation at ±1 °C every 60 s with a sampling interval of 1 s per point, and then heating to 25 °C at 3 °C/min. The Universal Analysis 2000 software (TA Instruments, New Castle, New Jersey, USA) determined the glass transition temperature. Large exothermic dips in heat flow identified the temperature of any crystallization events.

### SEC-HPLC

Lyophilized and stored samples were reconstituted with 200 μL of ultrapure water (NIBSC, Hertfordshire, UK), centrifuged at 13,600*g* for 5 min, and loaded onto a chilled autosampler. A 25 μL sample was injected onto a 7.8 × 300 mm, 5 μm particle size TSKgel G3000SWXL SEC-HPLC column (Tosoh Bioscience, Redditch, UK) on an Agilent 1200 workstation (Agilent Technologies, California, USA). G-CSF was eluted as monomer (19.9 min), dimer (18.4 min), or aggregate (10–12.5 min), with a 0.1 M phosphate pH 2.5 mobile phase at 1 mL/min, and peaks were monitored by absorbance at 214 and 280 nm and then integrated in Agilent ChemStation software (Agilent Technologies). All samples were measured in triplicate against a control buffer blank. Peaks were identified by comparison to analysis of a G-CSF reference standard (NIBSC, Potters Bar, UK).

Monomer retention (%) was calculated as
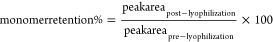
3

### Bioactivity

Samples were reconstituted for bioactivity assays as described above for monomer retention assays. An earlier procedure^23^ was modified to determine G-CSF potency as described previously.^20^ GNFS-60 cells were grown in T75 flasks (Sigma-Aldrich, Gillingham, UK) at 37 °C for 2–3 days, in 20 mL of RPMI 1640 Medium (Sigma-Aldrich, Gillingham, UK), with 2 ng/mL r-HuGCSF (Amgen, Uxbridge, UK), 0.5% (v/v) penicillin–streptomycin (Sigma-Aldrich, Gillingham, UK), and 5% (v/v) fetal bovine serum. At exponential growth, GNFS-60 cells were triple-washed by centrifugation at 250*g* for 10 min and resuspension in 20 mL of RPMI 1640 medium and then counted with a Countess Automated Cell Counter (Invitrogen, Life Technologies Corp, Paisley, UK). Cell viability was determined from 1:1 addition of 0.4% Trypan blue (Sigma-Aldrich Co, UK) at RT, which was then added immediately to a cell counting chamber slide with two 10 μL chambers. Cells for the bioassay were resuspended to 2 × 10^5^ cells/mL in RPMI 1640 medium. G-CSF samples, including the NIBSC second international reference standard for GCSF,^23^ were diluted to 4 ng/mL G-CSF in RPMI 1640 medium, and loaded as 100 μL per well of one row of a sterile 96-well plate (Falcon Microtest, Corning Life Sciences B.V., Amsterdam, Netherlands). RPMI 1640 medium was used to serially dilute samples into each new row, and then 100 μL of GNFS-60 cells was added to give 15.6–2000 pg/mL final G-CSF in each well. Covered plates were incubated at 37 °C for 48 h before addition of 20 μL of CellTiTer 96 AQueous One Solution (Promega, UK) and further incubation at 37 °C for 3–4 h. Absorbance at 490 nm was measured for each well in a plate reader (SPECTRAmax 340PC, Molecular Devices LLC, Wokingham, UK), with 5 s of shaking before reading, to determine GNFS-60 cell proliferation.

## Results and Discussion

### ssHDX-MS of Lyophilized G-CSF Formulations Containing Different Excipients

Our aim was not to probe the stability of the known Filgrastim formulation but rather to examine the effects of common excipients on protein stability using G-CSF as a model. Sorbitol is used in filgrastim, sucrose and mannitol are common excipients in both liquid and freeze-dried biologics, while amino acids are increasingly being explored, including in our previous liquid formulation studies for G-CSF.^18^ Using the same excipients would enable comparisons to those of our liquid formulation studies. Formulations of 0.3 mg/mL G-CSF in 50 mM citric acid, pH 4.25, with and without the addition of 1% (w/v) sucrose, 1% (w/v) mannitol, or 1% (w/v) phenylalanine, were compared by ssHDX-MS. In initial scale-down formulation screens in microplates (data not shown), we trialed these excipients and also sorbitol up to 3.5% (w/v), in three different buffers, before designing the final runs for HDX. Sorbitol was abandoned as the cakes collapsed under all conditions, while the other excipients did not. This was probably due to the low *T*_g_′ of −43 °C, compared to our primary drying at −45 to −25 °C, which avoided very long drying runs. 1% w/v was chosen for all excipients for comparability and set based on our previous studies in which phenylalanine quickly lowered the *T*_m_ at above 1% (w/v) for liquid G-CSF formulations.^18^

Citric acid was selected for G-CSF ssHDX-MS as it always produced a solid lyophilized cake in the scale-down formulation screens, which is required for robust comparisons with excipient-containing formulations. During preparation, new formulations were fully equilibrated by incubation at RT for 1 h, and then lyophilized in 2 mL glass vials, to form the cakes shown in Figure 2A. These were all white, structurally sound, and slightly shrunk back from the sides of the vial.

For deuterium exchange labeling, lyophilized vials were placed into sealed desiccators containing D_2_O and a RH of 43%, allowing labeling for 30, 60, 120, and 240 min. For each formulation, five unlabeled G-CSF sample vials were stored at −70 °C and used for HDX-MS of undeuterated samples, and three were stored at −20 °C prior to SEC-HPLC. Vials of samples labeled at each time point were removed, stoppered, and snapped frozen to quench exchange, avoiding submergence of the top of the vial to ensure liquid nitrogen could not enter.

**Figure 1:**
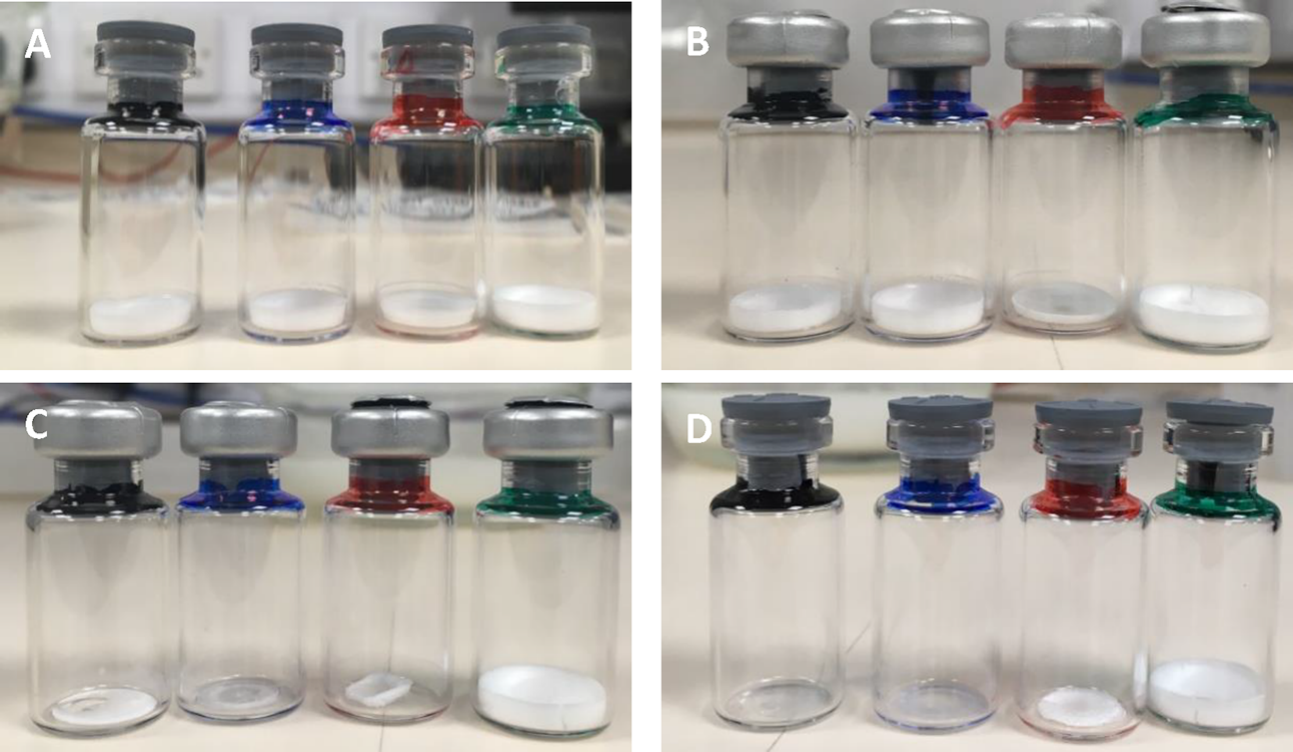
Appearance of lyophilized cakes during deuterium labeling in a desiccator at 43% RH. Vials contained lyophilized 0.3 mg/mL G-CSF in 50 mM citric acid buffer pH 4.25 and excipients denoted by the vial neck colors, where black contained no excipient, blue contained 1% sucrose (w/v), red contained 1% mannitol (w/v), and green contained 1% phenylalanine (w/v). Samples were labeled for (A) 0 min, (B) 60 min, (C) 120 min, and (D) 240 min. For simplicity, vials at 30 min labeling are not shown.

Cake appearance was monitored for each time point of labeling (Figure 1A–D). Cakes shrank back from the vial edges for the control and mannitol- and sucrose-containing samples, leading to loose but intact cakes. For phenylalanine, the cake cracked at the top but remained fixed to the bottom and edges of the vial. For the 30 and 60 min labeling in the 43% RH desiccators, all cakes remained visually unchanged. However, the control, mannitol, and sucrose samples had noticeably shrunk after 120 min with the onset of collapse. By 240 min, the control and sucrose cakes had collapsed. The phenylalanine-containing cake did not change throughout labeling. Based on the appearance of the cakes, the HDX analysis focused on comparisons at the 120 min time point. Therefore, the HDX analysis was reporting on exchange in the solid state but also as it absorbed water vapor up to a semisolid state prior to collapse.

### HDX-MS

The process of sample defrosting, reconstitution, and mixing with cold quench solution to the point of LC-MS injection took an average time of 1 min 52 s. Peptide mapping using the HDX-MS protocol with no exchange showed that the coverage of lyophilized and reconstituted G-CSF was 97.7% (Supporting Information, Figure S1). The general impact of a wider set of excipients on HDX uptake rates was also evaluated by using formulations containing an internal reference peptide (IRP). This found that G-CSF had significantly lower uptake after 2 h for arginine and glycine (Supporting Information, Figure S2). By contrast, G-CSF in mannitol and phenylalanine had the same uptake as the control with no excipient. Uptake for G-CSF in sucrose was also lower than that in the control sample, although this difference decreased at the 4 h time point and after cake collapse. Therefore, any overall changes in uptake for G-CSF formulations in mannitol and phenylalanine can be directly attributable to their influence on the G-CSF structure, whereas for sucrose, a general contribution from the surrounding medium also needs to be accounted for.

The overall mass of exchange summed for all G-CSF peptides at different labeling time points, expressed as a percent of the maximum possible exchange, is shown in Table 2. At 30 min, the overall mass exchanged was still low and sucrose data was not available at this time point. At 120 min, the excipients were clearly protecting with sucrose the most protective, exchanging 7.2% of G-CSF peptide protons compared to 12.9% in the control sample. For comparison, phenylalanine also protected well with 8.2% exchanged and mannitol less well with 10.3% exchanged. The same result was seen at the 420 min time point, with sucrose still the most protective, followed by phenylalanine and then mannitol. However, due to the collapse of the control sample observed at 240 min (Figure 1D), the interpretation of exchange after 420 min of labeling is more complex and so only the earlier time points were used for further analysis.

**Table 2:** Total Exchange as % of Maximum Exchangeable Peptide Protons for All Lyophilized Formulations

	**total exchange(% of max)**		
**excipient**	**30 min**	**120 min**	**420 min**
**none (control)**	2.7	12.9	30.6
**sucrose**	N/A	7.2	21.9
**mannitol**	7.6	10.3	29.3
**phenylalanine**	9.0	8.2	26.0

Peptide-level fractional exchange plots for all time points up to 420 min are shown in the Supporting Information (Figure S3). From these, it was clear that 30 min of exchange was just able to detect and quantify the exchange in all regions, but the signal-to-noise was much improved after 120 min of exchange. The increase in exchange was exponential as expected, and so at 240 min in the control sample, the exchange was beginning to saturate with only a slight further increase in exchange to 420 min. Taken together with the onset of cake collapse evident at 120 min, and in some cases complete at 240 min (Figure 1D), the optimal exchange time to analyze was at 120 min, as the kinetics were still close to linear (50% of the exchange reached at 420 min, and up to 30% of the maximum possible exchange for some peptides).

Exchange broadly occurred in loop regions and the more solvent-exposed sections of the helices. Exchange kinetics were measurable on 72% of the G-CSF structure, with the remainder being too protected from exchange, and located mainly in the most stable helical regions including the central regions of helices A, B, and D, as well as the C-terminus of helix C. This degree of protection was slightly higher than in previous HDX studies for liquid formulations of G-CSF,^17,18^ for which exchange was also observable throughout helix D. This highlights that for G-CSF in the lyophilized solid state, the dynamics of the protein were distributed similarly to those in the liquid state, except for helix D that became more protected in the solid state. Thus, freeze-drying did not significantly alter the overall topology of G-CSF, although it may have led to increased stabilization or self-interaction via helix D.

**Figure 2:**
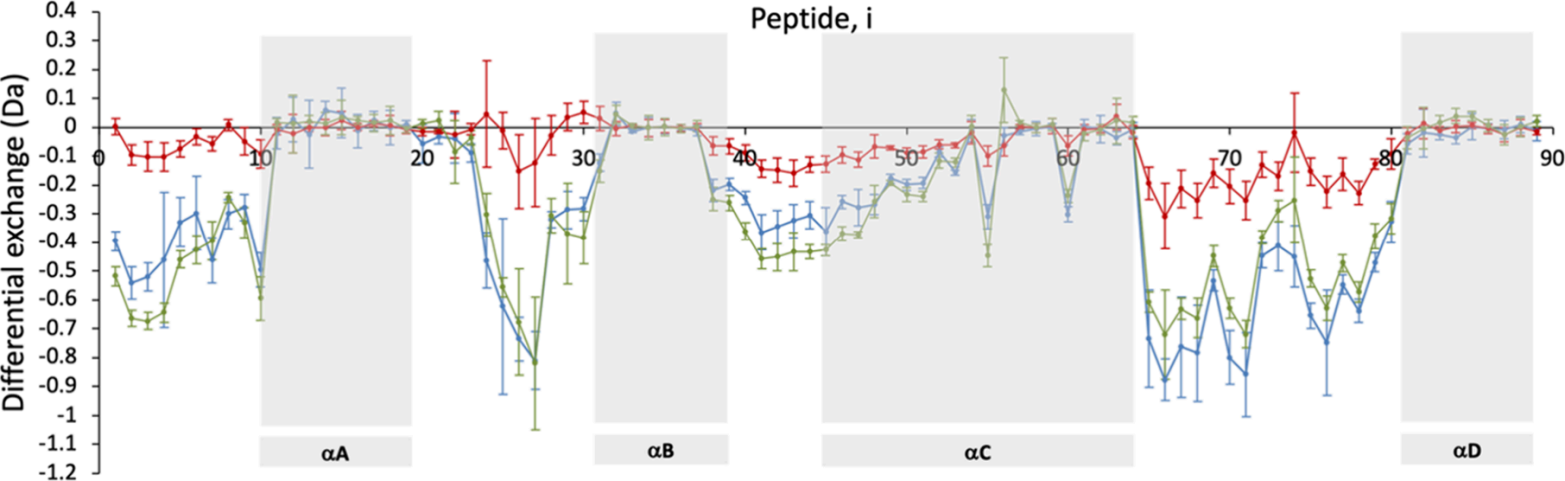
ssHDX-MS differential exchange plots of peptides for G-CSF lyophilized in each excipient formulation. Deuterium uptake values were taken from G-CSF peptide-level ssHDX-MS with 0.3 mg/mL G-CSF lyophilized in 50 mM citric acid, pH 4.25, 1% w/v excipient, with 120 min exchange against D_2_O at 43% RH. The *y*-axis denotes the differential uptake relative to the control with no excipient, Δ*D*(*t*) = *m*_excip_ – *m*_cont_, where *m* denotes the absolute mass change of the peptide after exchange, for (blue) sucrose, (red) mannitol, and (green) phenylalanine. Negative values indicate a decrease in uptake in the presence of the excipient. The *x*-axis labels the identified peptides 1–89 of G-CSF common to all experiments, ordered according to their midpoint residue. The different helical regions of G-CSF are highlighted as gray boxes in the background. The noncolored regions represent the connecting loop regions.

The peptide-level differential (excipient control) exchange after 120 min, for each of the three G-CSF lyophilized formulations relative to the control without excipients, are shown in Figure 2. It is clear that sucrose gave more negative values overall and so was the most protecting from exchange in the solid state, especially in the long loopCD. Phenylalanine was only slightly less protective overall but was more protective than sucrose in some specific regions such as at the N-terminus, and in part of loopBC. Overall protection by sucrose and phenylalanine was more extensive than previously in the equivalent liquid formulations of G-CSF and tended to protect across most regions for which exchange was significant in the control sample. However, regions with significant absolute exchange but low differential exchange included peptides 20–22 (start of loop AB), peptide 31 (start of helix B), peptides 49–54 (end of loop BC and start of helix C), and peptide 81 (start of helix D). Thus, while sucrose and phenylalanine were mapped previously to two specific interaction sites on G-CSF in liquid formulations, there may be one or more additional sites with protective effects in the dried solid state. It is also possible that lyophilization leads to a switch toward a global but nonuniform stabilization of G-CSF through a preferential exclusion mechanism. However, water is removed from the protein during drying, concentrating solutes in solution in a way that is more likely to promote protein-excipient interactions.

By comparison, mannitol provided considerably less protection overall, typically at 25–50% of that provided by sucrose. A few regions protected well by sucrose and phenylalanine were not protected at all by mannitol, including in the first half of loopAB. This suggests possible regions that interact with sucrose and phenylalanine but not mannitol and points away from a preferential exclusion mechanism with mannitol at least. This may have been driven by the known tendency of mannitol to recrystallize during lyophilization. It is also possible that the differences lie in regions that form protein–protein interactions within a dimer or aggregate that are abrogated only by mannitol. Mannitol also potentially increased the exchange in the last portion of loopAB, a region into which it was predicted previously to bind in solution,^18^ although the differential increase in the lyophilized G-CSF was close to the limits of the associated error bars. This increase was not seen previously in the equivalent liquid formulation and so may represent a minor change in structure due to being lyophilized.

### Monomer Retention by SEC-HPLC

**Figure 3:**
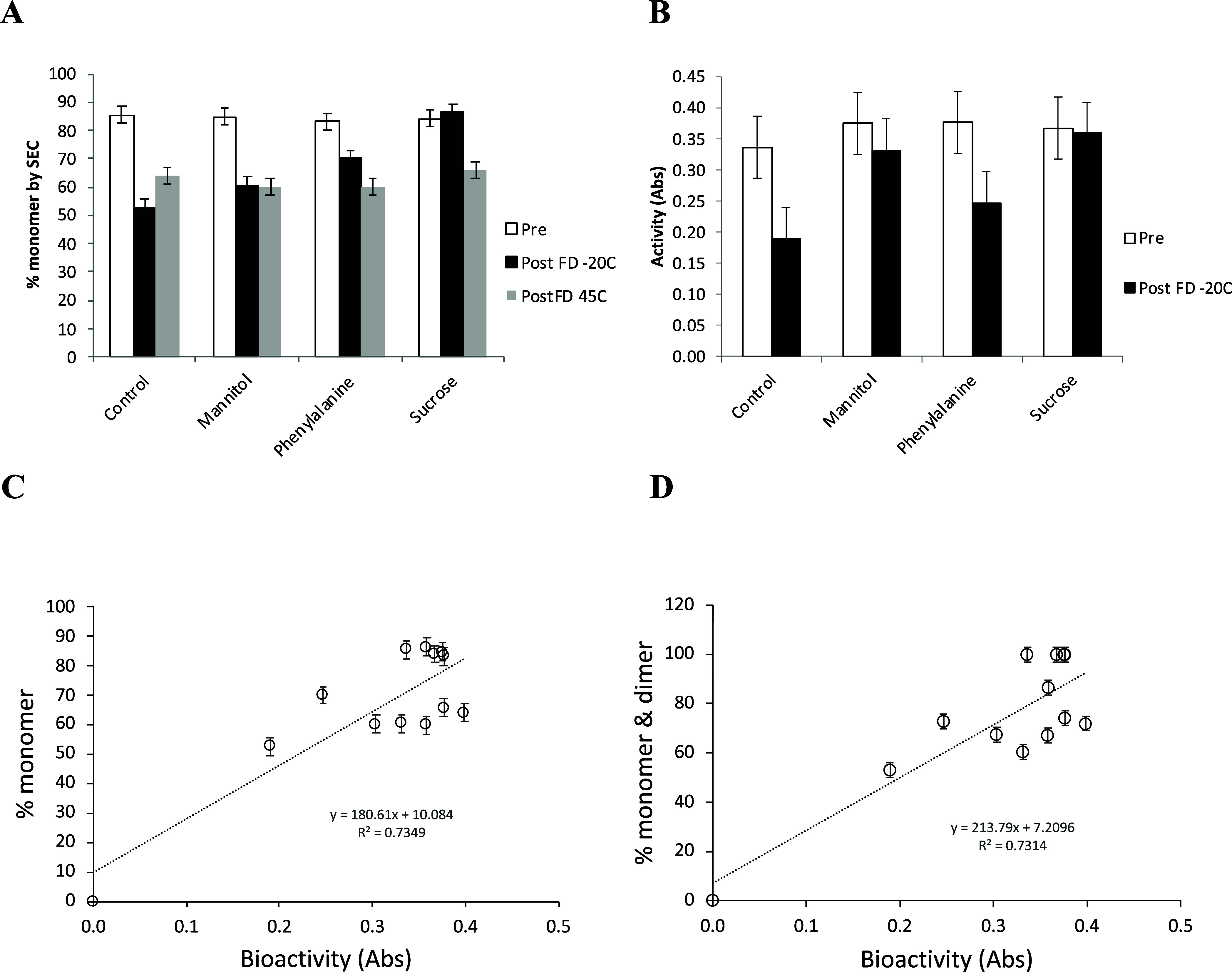
Impact of excipients on monomer content and bioactivity of G-CSF with mannitol, phenylalanine, and sucrose pre- and post-lyophilization. (A) Monomer content determined by SEC before (white) and after lyophilization and storage at −20 °C (black) or at 45 °C (light gray) for 30 days. (B) Bioactivity of G-CSF before (pre) and after (post −20 °C) lyophilization and storage at −20 °C for 30 days. Correlations between bioactivity and (C) monomer only or (D) monomer plus dimer content. Samples contained 0.3 mg/mL G-CSF in 50 mM citric acid pH 4.25, with no excipient (control), or either 1% (w/v) mannitol, phenylalanine, or sucrose. Measurements were taken from three independently processed samples, and error bars denote standard deviations.

The content of G-CSF monomer, dimer, and larger aggregates, before and after lyophilization when formulated with sucrose, mannitol, or phenylalanine, compared to the no-excipient control was determined using SEC-HPLC. Postlyophilization samples were stored at both −20 and 45 °C for comparison prior to analysis by SEC-HPLC. All samples started with 84% monomer content, 16% dimer, and no higher-order oligomers (Supporting Information, Figure S4). Lyophilization with 30-day storage at −20 °C led to complete depletion of the dimer for all samples but varying degrees of monomer loss and formation of aggregate. The control sample ended with only 53% monomer and 0.4% dimer, indicating 46.5% aggregate as determined from the loss of total peak area, giving a retention of 62% ± 4% of the original monomer. The excipients all stabilized the monomer against losses during lyophilization and storage at −20 °C (Figure 3A) with sucrose the most stabilizing, retaining 102% ± 5% of the original monomer. Phenylalanine was the second most stabilizing with 84% ± 5% of the original monomer retained, while mannitol retained 71% ± 4% of the original monomer. Thus, the control samples and then mannitol led to the most aggregation with lyophilization and storage at −20 °C, while sucrose and then phenylalanine were the most stabilizing to monomer loss.

None of the excipients could avert depletion of the dimer. Dimers of G-CSF have been observed previously as an irreversible disulfide-bonded form on the pathway to aggregation in solution conditions,^24^ but also as a reversible form under physiological conditions, which is not directly involved in aggregation.^25^ Therefore, dimer depletion could potentially occur through reversible dissociation to monomer, maintaining [dimer]/[monomer]^2^ when monomer is converted into aggregate, as was demonstrated previously in sucrose-containing solutions.^25^ However, this does not explain the loss of dimer during lyophilization in the presence of sucrose where the monomer content is maintained. Alternatively, the dimer could be inherently more aggregation-prone than the monomer during lyophilization, leading to its loss in all formulations.

Earlier it was noted that HDX experiments indicated a few regions, including the first half of loopAB, that were protected by sucrose and phenylalanine but not by mannitol. One possible explanation was that these regions might be protected from exchange through protein–protein interactions within the dimer for sucrose and phenylalanine but not by mannitol. The maximum dimer content was 16%, and only at the start of HDX, which would lead to some level of protection in certain peptides, but only for 16% of the total sample. However, the level of protection in sucrose or phenylalanine for some peptides was >40% compared to that in mannitol, and so the dimer could only be a minor contributor. Alternatively, the protection may be due to protein–protein interactions within aggregates for sucrose and phenylalanine only. However, the SEC analysis found that mannitol led to higher aggregate content, which would have been expected to lead to the opposite result of increased HDX protection for mannitol. Thus, it was more likely that these regions were protected through direct interactions with sucrose and phenylalanine, but not with mannitol.

We also examined the stability of lyophilized samples to 30-day storage at 45 °C using SEC-HPLC. Interestingly, the end results were similar for all samples with approximately 60–66% ± 3% monomer content after 45 °C storage, equivalent to 71–78% ± 4% retention of the starting monomer content. Therefore, while sucrose and then phenylalanine were more protective than mannitol and the control against the lyophilization process itself (including storage at −20 °C), the gains made in the lyophilization step were subsequently lost through aggregation within the dried cake stored at 45 °C.

One difference at 45 °C was that the dimer content did not remain close to zero after lyophilization as for −20 °C, but instead all samples ended with 7–8.5% ± 2% dimer (Supporting Information, Figure S4). Therefore, heating to 45 °C promoted dimer formation in lyophilized cakes. G-CSF is known to undergo a structural conformation change at above 37 °C in the liquid state, as observed by NMR.^26^ Such a shift in structure could potentially promote dimer formation in the dried state, but this has not been investigated here.

### Bioactivity

The retention of bioactivity followed closely the retention of G-CSF monomer as seen in Figure 3B and their direct correlation in Figure 3C, which had an *R*^2^ of 0.73 for pre- and postlyophilization at −20 and 45 °C combined. However, the experimental error associated with variability in the bioactivity assay was significantly larger than that for SEC-HPLC making it a less robust indicator of sample preservation. The 45 °C stored samples clustered slightly separately on the plot. As the 45 °C samples also contained significantly more dimer, we combined the monomer and dimer content together (Figure 3D), but this did not improve the correlation with bioactivity. This suggested that the dimer in 45 °C samples did not contribute significantly to bioactivity, and is further evidence that in these conditions the dimer was not reversibly formed.

### Glass Transition (*T*_g_′) and Crystallization Temperatures in the Frozen State

Modulated DSC was used to further characterize the formulations by determining their glass transition (*T*_g_′) and crystallization temperatures in the frozen state, based on measurements of total, reversible, and nonreversible heat flow (Table 3). The *T*_g_′ values are likely to have been influenced by a combination of the excipient content and also the residual moisture content of the dried cakes. Overall, the formulations did not significantly affect *T*_g_′ such that the control and mannitol- and sucrose-containing samples had *T*_g_′ values (total heat flow) of −33.6, −38.3, and −33.5 °C, respectively. Mannitol also showed a slight crystallization transition in the reversible heat flow at approximately −15 °C. Phenylalanine did not give a measurable *T*_g_′ but showed a crystallization transition in the reversible heat flow at −12.1 °C. The results indicate that the majority of the formulations form amorphous solids but that more crystalline material is formed with phenylalanine while mannitol was amorphous mixed with a small amount of crystalline material. However, these results also indicate the potential for phenlylalanine and mannitol to recrystallize further during lyophilization and storage. Again, this may be the main reason for which mannitol was observed to have less protection from HDX in the solid state.

**Table 3:** Modulated DSC and Freeze-Drying Microscopy for G-CSF with Different Excipients^a^

	**DSC***T*_**g**_′ (°C)			**FDM***T*_**c**_**(°C)**
**excipient**	**heat flow**	**rev heat flow**	**nonrev heat flow**	**collapse** (±0.1)
none (control)	–33.5 (0.2)	–32.1 (0.02)	–33.7 (0.2)	–33
sucrose	–33.5 (0.1)	–31.4 (0.1)	–33.3 (0.1)	–31
mannitol	–38.0 (0.5)	–35.1 (1.8)	–38.3 (0.25)	–35.6
phenylalanine		–12.1 (0.2)		–19

a Samples of 0.3 mg/mL G-CSF in 50 mM citric acid, pH 4.25, were analyzed in triplicate, and thermal events were determined from the total heat flow, reversible heatflow, and nonreversible heatflow by DSC and from the collapse observed by freeze-drying microscopy. Standard deviations (*n* = 3) are shown in parentheses. FDM values were obtained only once; the error of ±0.1 °C is based on the resolution of temperatures between image frames.

FDM was also used to evaluate the collapse temperatures of the formulations (Table 3). These correlated with the *T*_g_′ for the control, mannitol, and sucrose formulations and with the crystallization temperature for phenylalanine. However, the *T*_g_′ and collapse temperatures did not correlate with the observed HDX or monomer loss during lyophilization. This confirmed the appropriate selection of −45.0 °C for the freezing step used during lyophilization, which avoided any influence of cake collapse or glass transition on the stability and exchange kinetics of G-CSF formulations.

### Relationship between HDX Kinetics and Monomer Loss

**Figure 4:**
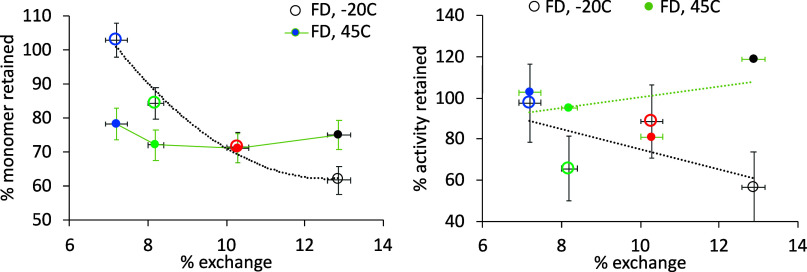
Total hydrogen–deuterium exchange vs monomer and activity retained after freeze-drying. Overall less stable G-CSF formulations lead to greater fraction of the possible exchange within 120 min. Formulations are color coded for (black) control, (blue) sucrose, (red) mannitol, and (green) phenylalanine. Errors are standard deviations for three repeats (monomer retained) or four to five repeats (% exchange).

The monomer losses from SEC-HPLC, and the corresponding bioactivity losses for all formulations, were each compared to the total exchange for all peptides expressed as a percentage of maximum possible exchange (Figure 4). An almost linear trend was observable between total exchange and the monomer content remaining after lyophilization with storage at −20 °C. Sucrose was the most protective to G-CSF as measured by both HDX kinetics in the solid state and the loss of monomer during lyophilization (stored at −20 °C), while phenylalanine, mannitol, and the control were progressively less protective. The same trend was observable, though noisier, when measured by loss of bioactivity (Figure 4B). Therefore, the total differential uptake was an effective predictor of stability against the lyophilization process and subsequent storage at −20 °C, or at least provided an effective measure of the monomeric state of the protein in the lyophilized cake.

This result was consistent with aggregation mechanisms observed previously in liquid formulations, in which the native structure is an ensemble of states in a rapid dynamic equilibrium and that at least one of those states, designated N*, is partially unfolded to reveal an aggregation-prone region. High exchange seen by HDX in structured and/or buried regions is indicative of rapid fluctuations in the native ensemble, and conditions that promote exchange are also more likely to promote the formation of N* and in turn increase aggregation kinetics. As outlined above, G-CSF aggregation kinetics in liquid formulations fitted best to models, which assumed that partial unfolding (e.g., to N*) was rate limiting.^19^ We have also shown previously that HDX correlated very well to aggregation kinetics in liquid formulations for a series of G-CSF variants and identified a probable N* state in which loop AB and loop CD were structurally altered to partially reveal a region of helix B and the beginning of loop BC.^18^ Further below, we investigate whether the N* state is similar for G-CSF aggregation in the lyophilized state.

For lyophilisates stored subsequently at 45 °C, there was little difference in final monomer content between any of the formulations, as measured by either SEC-HPLC or bioactivity, and so there is no direct relationship with total HDX. This is perhaps unsurprising as the HDX was carried out on lyophilized cakes at 22 °C and so would not necessarily identify any changes in structure and subsequent (de)protection that occurred specifically at 45 °C after lyophilization. Thus, overall, sucrose, and to a lesser extent phenylalanine, provided protective mechanisms that stabilized structural dynamics at 22 °C and minimized bioactive protein loss during lyophilization and storage of the dried cakes at −20 °C, but these protective mechanisms were lost when the lyophilized protein was stored at 45 °C. Thus, the dominant mechanisms of monomer loss at −20 and 45 °C appeared to be different. The most likely explanation is that incubation at 45 °C led to a new structural conformation and/or aggregation mechanism that was not dependent on the structural dynamics observed by HDX at 22 °C and that the monomer losses relating to this change were no longer affected by the presence of any of the excipients. This observation is also consistent with the structural conformation changes in G-CSF observed by NMR at above 37 °C in the liquid state,^26^ but again any structural difference at 45 °C is not directly investigated here.

### Mapping HDX at the Structure Level to Monomer Loss

**Figure 5:**
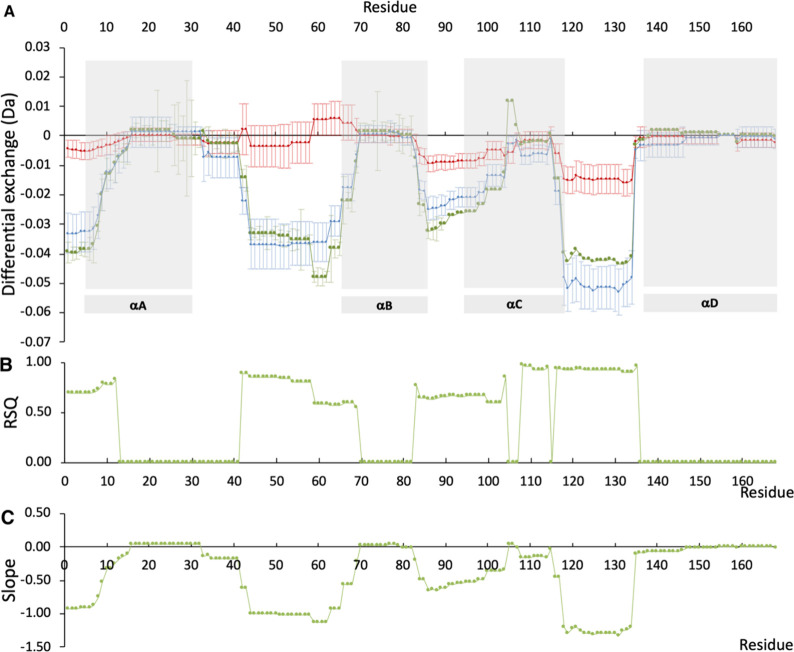
Residue-level exchange and correlation to monomer loss for lyophilized G-CSF formulations. (A) Residue-level differential exchange determined from weighted average of contributing peptides, for each formulation with (blue) sucrose, (red) mannitol, and (green) phenylalanine, relative to the control. (B) RSQ and (C) slopes (×1000), from linear correlations between residue-level absolute exchange and monomer loss after lyophilization and storage at −20 °C.

The peptide-level exchange shown in Figure 2 as differential uptake from the control samples was converted to a pseudoresidue level using a weighted average of the exchange for all peptides that contain each amino acid (Figure 5A). The relationship between residue-level exchange and the monomer loss after lyophilization and storage at −20 °C was then investigated, by linear correlations for the four formulations. It must first be noted that the HDX experiment does not report well on regions with low absolute exchange in all samples and that these inevitably lead to low RSQ and slopes for linear correlations. Such regions include central regions of helices A, B, and D, as well as the C-terminus of helix C as discussed above.

Regions with significant absolute exchange but low differential exchange would also give low RSQ and slopes. This occurs in peptides 20–22 (residues 33–41, start of loop AB), peptide 31 (residues 63–69, end of loop AB and start of helix B), peptides 49–54 (residues 89–103, end of loop BC and start of helix C), and peptide 81 (residues 135–146 at the start of helix D) and indicates that the exchange in these regions is not significantly influenced by the excipients tested and so G-CSF is unlikely to have undergone any structural changes in these regions that

could affect monomer loss. The remaining regions with significant absolute exchange and high differential exchange indicate a significant impact of the excipients tested on local structure and dynamics either directly or indirectly. For these regions, the values of RSQ and slopes from linear correlations (Figure 5B,C) identify structural changes, of which some may directly impact on monomer loss. We considered only values of slopes or RSQ at residues for which their differential exchanges exceeded at least 1.5× their standard deviations. In this way, the slopes and RSQ values at those residues can be considered to be statistically robust.

**Figure 6:**
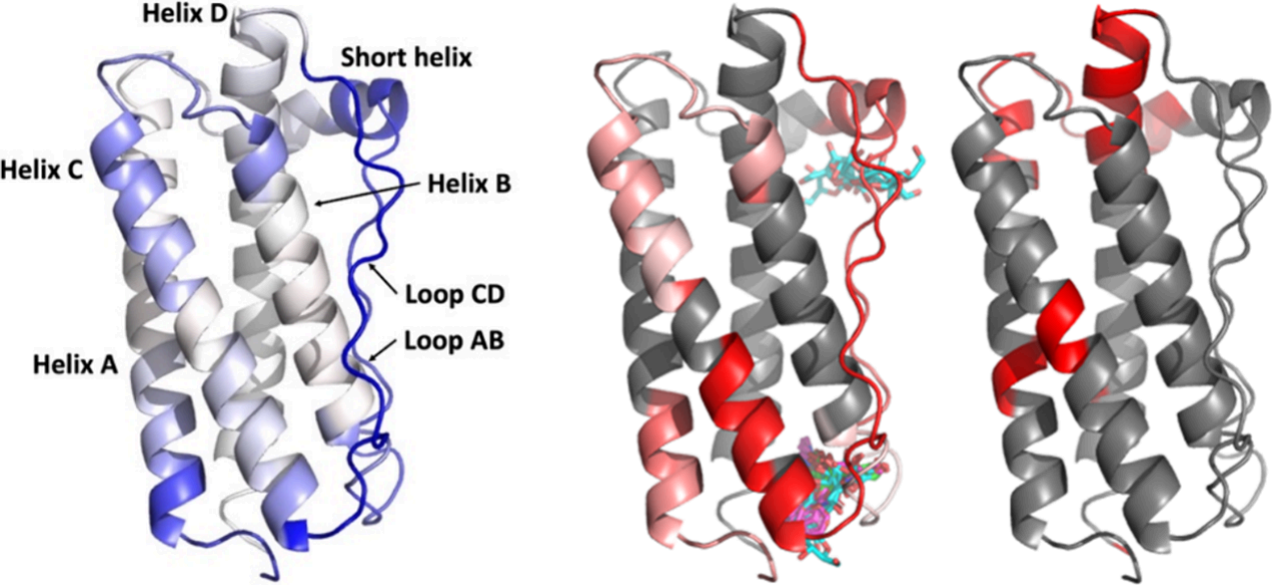
Residue-level structure heat map of RSQ from monomer protection in FD vs residue HD exchange at 120 min. (A) Slopes from FD protection vs HDX (blue strong negative slope). (B) *R*^2^ of residues where differentials exceed 1.5σ (gray not significant, RSQ = 0 white, RSQ = 1 red). Sites of excipient binding predicted by docking are also shown with sucrose (cyan), phenylalanine (magenta), and mannitol (green) shown in all top 10 docking poses. (C) Sites with significant exchange rates (>1.5σ) but no significant differential with excipients. Images were generated in PyMOL Molecular Graphics System (Schrödinger, USA) using the G-CSF crystal structure PDB ID: 2D9Q.^27^

The significant slopes and RSQ values for linear correlations between HDX and monomer loss during lyophilization were mapped to the structure of G-CSF, as shown in Figure 6. Regions with significant slopes and high RSQ (all >0.57) were broadly the same. Thus, stabilization by excipients was clearly clustered in the N-terminus of helix A (*R*^2^ 0.73), the short helix and loop AB (*R*^2^ 0.84), and then the C-terminus of helix C and loop CD (*R*^2^ 0.91). Slightly weaker correlations were observed in the final bend of loop AB (*R*^2^ 0.58), and the region spanning the C-terminus of helix B to the N-terminus of helix C (*R*^2^ 0.66). All other regions gave no significant change in exchange, resulting from the presence of excipients. Of these, some regions gave no statistically significant change (differential) but still had significant absolute exchange rates (above 1.5 sigma). These included a structural cluster comprising the C-terminus of helix A, through the start of loop A and the first turn of the short helix and then the N-terminus of helix D. Also a separate band of residues connecting three residues each in the middles of helices A and C, with residue Y159 near the end of helix D (Figure 6C). These regions represent a structure that did not respond to the presence of excipient as measured by HDX. It therefore appears that these regions were not critical to controlling monomer loss during freeze-drying.

Unobservable regions (with no significant exchange in any sample after 120 min) included most of the middle and C-terminal half of helix D, the central two-thirds of helix B, and 10 residues spanning the middle and the last half of helix A. These regions were the least dynamic and most stable and therefore least likely to be directly involved in stabilization by excipients. However, helix D does form the major aggregation-prone region (APR) as previously predicted^26^ using the AmylPred2 consensus approach,^28^ and so the dynamics and unfolding of surrounding structure, mainly of loop AB and the short helix, could be critical to its exposure.

Overall, the results show that tolerance to freeze-drying (stored −20 °C) was gained mainly through decreased dynamics and increased stability in the small helix, loop AB, helix C, and loop CD. The loop AB and loop CD regions in particular match those observed previously as critical to aggregation and conformational stability in liquid formulations of G-CSF, as monitored by LC-MS HDX and NMR,^18,26^ while the role of loop AB in the aggregation pathway was also inferred through the previous observation of hyperfluorescence.^24^ Previously, the HDX and aggregation kinetics also correlated very well for liquid formulations of the G-CSF variants. Detailed mapping of HDX revealed that the aggregation-prone state N* was most likely to involve remodeling of a core region involving loop AB and loop CD, exposing helix B and the beginning of loop BC.^18^ Interestingly, the excipients sucrose, mannitol, and phenylalanine were also predicted by docking and observed by changes in HDX in the liquid state to interact with G-CSF in at least two locations, notably at both ends of loop AB. Protection of the same sites in the lyophilized formulations indicates that interactions occur similar to those in the liquid formulations, in addition to some new sites. Previous thermal ramping of liquid G-CSF formulations monitored by NMR also showed structural changes at 37 °C within loop AB, loop CD, and small elements of helix C and helix D.^26^

## Conclusions

The stabilization of G-CSF with different excipients during lyophilization was studied in the solid state using HDX-MS to investigate the role of structural dynamics in maintaining stability of G-CSF with different excipients in the solid state during lyophilization. This also enabled a comparison with our previous HDX-MS analysis for liquid formulations to determine whether the critical regions of the structure that led to aggregation were similar. The 43% relative humidity required for vapor-phase D_2_O exchange were found to induce cake collapse in lyophilized G-CSF samples after prolonged exchange times. Fortunately, sufficient exchange had already occurred prior to collapse to obtain a complete and comparable analysis by ssHDX-MS in all formulations. Global exchange levels in freshly lyophilized G-CSF formulations at 22 °C correlated well with the stability of G-CSF based on the residual monomer content measured after lyophilization and storage at −20 °C. This was consistent with previous studies by ssHDX of other proteins and confirmed the ability of ssHDX to predict shelf life over longer periods. Sucrose gave the greatest protection, with phenylalanine, mannitol, and the no-excipient control having progressively less protection and also storage stability. This result was in keeping with previous ssHDX-MS studies^11^ where both global and peptide-level ssHDX was lower in the presence of sucrose than with mannitol, for equine myoglobin (Mb) formulations. Sucrose is a well-known cryoprotectant forming a stabilizing amorphous matrix. By contrast, mannitol often transitions to crystalline forms, while phenylalanine was observed here to have a crystallization transition. Such crystalline or partially crystalline forms may not provide adequate stabilization to the protein compared to amorphous matrices. Recent ssHDX studies found mannitol formulations to be structurally perturbed relative to those with sucrose and also to contain fewer hydrogen-bond interactions between the protein and surrounding matrix.^8,9^ The predictive power of ssHDX was lost for samples stored at 45 °C where there was little difference in the final monomer content between the formulations. We propose that the higher temperature led to a new structural conformation or aggregation mechanism, possibly related to a conformational change at 37 °C observed previously by NMR for liquid-formulated G-CSF. Analysis of ssHDX at the peptide-level revealed that increased protection in the small helix, loop AB, helix C, and loop CD was linked to improved stability for lyophilization with −20 °C storage. This structural protection was very similar to that found previously in stabilized liquid formulations, although it covered some additional regions in the solid-state indicative of more extensive stabilization in the solid matrix compared to that in solution. This could reflect greater conformational constraints in the solid state due to lower mobility within the surrounding matrix or more extensive interactions formed with excipients in the solid matrix than in solution.

## Supporting Information Available

Peptide map coverage for G-CSF HDX-MS; sequence coverage of non-deuterated G-CSF from pepsin-digest peptide mapping; excipient impact on relative deuterium uptake for a lyophilized internal reference peptide (IRP); time-course for peptide-level fractional exchange of 0.3 mg/mL G-CSF lyophilized in 50 mM citric acid pH 4.25 with 1% (w/v) excipients as measured by ssHDX-MS (PDF)
