# Supplementary material for: Investigation of the Solid-State Interactions in Lyophilized Human G-CSF Using Hydrogen–Deuterium Exchange Mass Spectrometry
Source: Mol Pharm. 2024 Mar 22;21(4):1965–76. doi: 10.1021/acs.molpharmaceut.3c01211 (PMC10988552; doi:10.1021/acs.molpharmaceut.3c01211)
Supplement: Supplementary file 1 — mp3c01211_si_001.pdf [file mp3c01211_si_001.pdf]

Supplementary Information:

Investigation of the solid-state interactions in lyophilised human G-CSF using hydrogen-deuterium exchange mass spectrometry

Victoria E Wood[1], Mark-Adam Kellerman[1], Kate Groves[3], Milena Quaglia[3]
Elizabeth M Topp[4], Paul Matejtschuk[2], Paul A Dalby[1]

1. Department of Biochemical Engineering, University College London, London, WC1E 6BT, UK

2. Standardisation Science, NIBSC, Medicines & Healthcare products Regulatory Agency, South Mimms, Hertfordshire, EN6 3QG, UK

3. LGC, Queens Road, Teddington, Middlesex, TQ11 0LY, UK

4. Department of Industrial and Molecular Pharmaceutics, College of Pharmacy, and Davidson School of Chemical Engineering, College of Engineering Purdue University, West Lafayette, Indiana 47907, United States

Peptide map coverage

Three injections of reconstituted non-deuterated control sample were used to generate a peptide map by LC-MS, and this gave a sequence coverage of 97.7% (Figure S1).

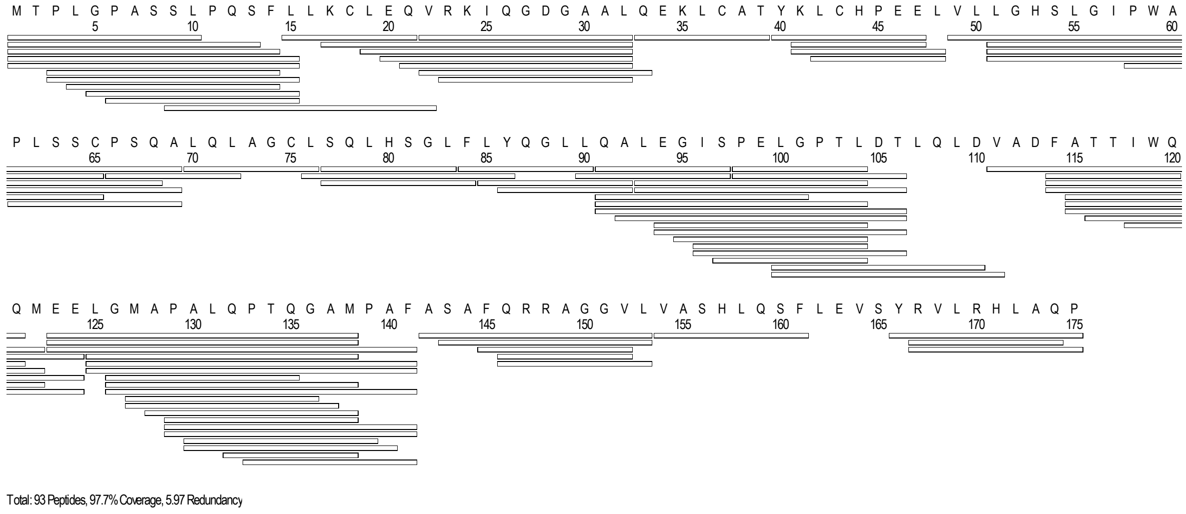

Figure S1. Sequence coverage of non-deuterated G-CSF from pepsin-digest peptide mapping. G-CSF at 0.3 mg/mL in 50 mM citric acid, pH 4.25 was lyophilised and reconstituted in 0.2% (w/w) formic acid at a 10x dilution. The peptide map was generated from 5 injections of the sample into the HDX-MS system following 1:1 mixing in quench solution. Peptides were identified using Waters PLGS excluding pepsin, with a minimum product per amino acid of 0.03, a maximum MH^+ Error (ppm) of 20 and a file threshold of 3.

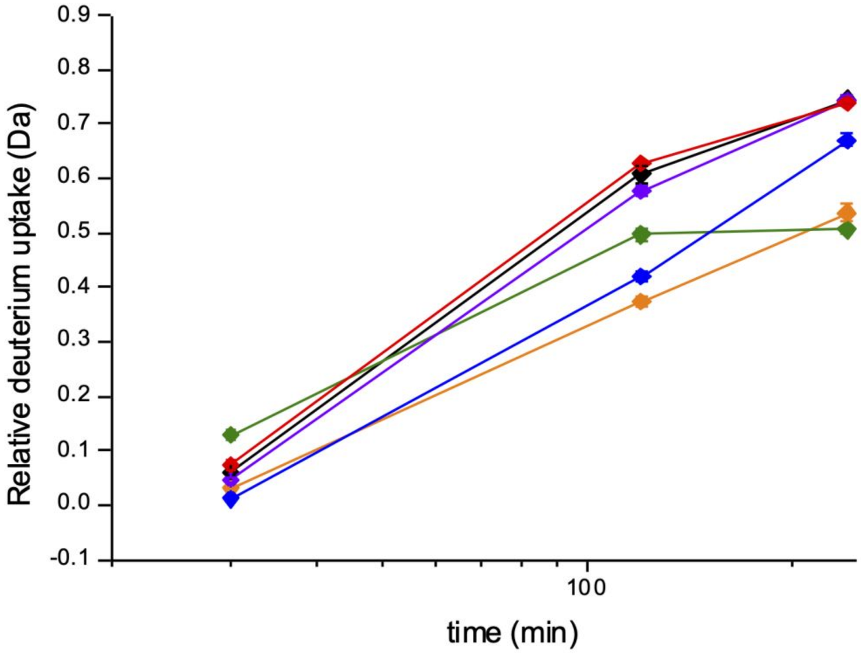

Figure S2. Relative deuterium uptake for internal reference peptide (IRP) lyophilised with different excipients. Samples were labelled at three different times: 30 mins, 2 hours, and 4 hours. All samples were formulated in 50 mM citric acid pH 4.25. Excipients studied at 1%(w/v) included arginine (orange), glycine (green), mannitol (red), phenylalanine (purple), sucrose (blue) and no-excipient control (black).

The relative deuterium uptake of an internal reference peptide (IRP) was evaluated with different excipients alongside a no-excipient control. At the earliest labelling time point (30 min), the uptake of D_2O was low for all samples apart from glycine. At the second labelling time point (2 hrs) the mannitol and phenylalanine samples were within the error of the control, whereas the arginine, sucrose and glycine samples had significantly lower exchange than the control. Finally, at the longest labelling time point (4 hrs) the mannitol and phenylalanine samples again followed the same uptake rate as the control, whereas the arginine sample remained lower than the control. The sucrose uptake came closer again to that of the control, though remained slightly lower. The glycine uptake plateaued, remaining at the same level that it had for the 2 hr time point. Based on appearance, all formulations generated white cakes, but after 2 hrs incubation the buffer only, mannitol and arginine cakes had shrunk slightly, while the glycine cake had disappeared i.e. fully reconstituted. This suggests that the glycine formulation took up moisture quicker than the others, hence the increase in exchange and early plateau. All other excipients, although their cakes collapsed at 4 hrs, still increased in uptake over time.

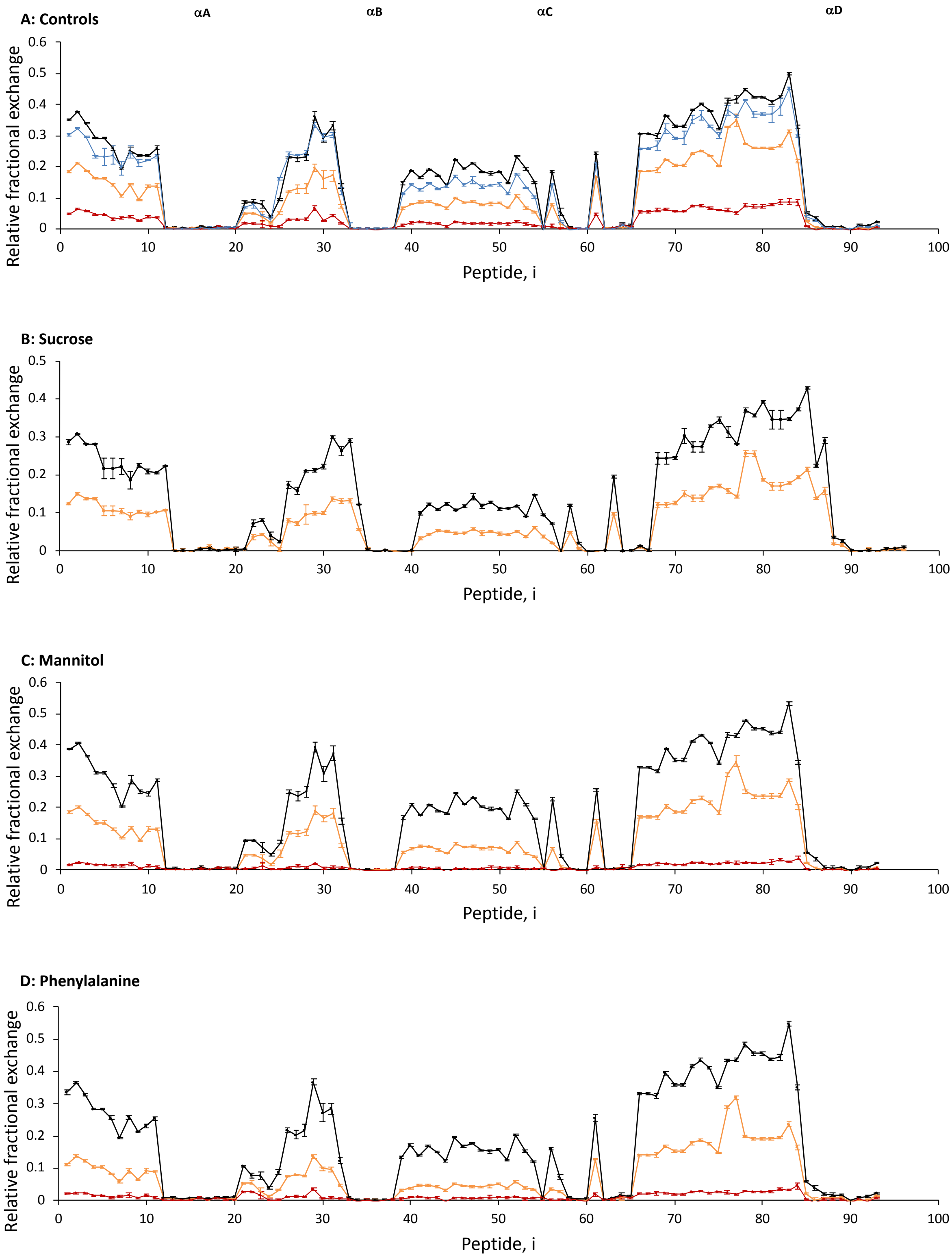

Figure S3. Time-course for peptide-level fractional exchange of 0.3 mg/ml G-CSF lyophilised in 50 mM citric acid pH 4.25 with 1%(w/v) excipients as measured by ssHDX-MS. Relative fractional exchange was measured at 30 mins (red), 120 mins (orange), 240 mins (blue), and 420 mins (black). The peptide number (i) in the x-axis represents the 93 overlapping peptides obtained from pepsin digestion. The helix locations are denoted in the top figure.

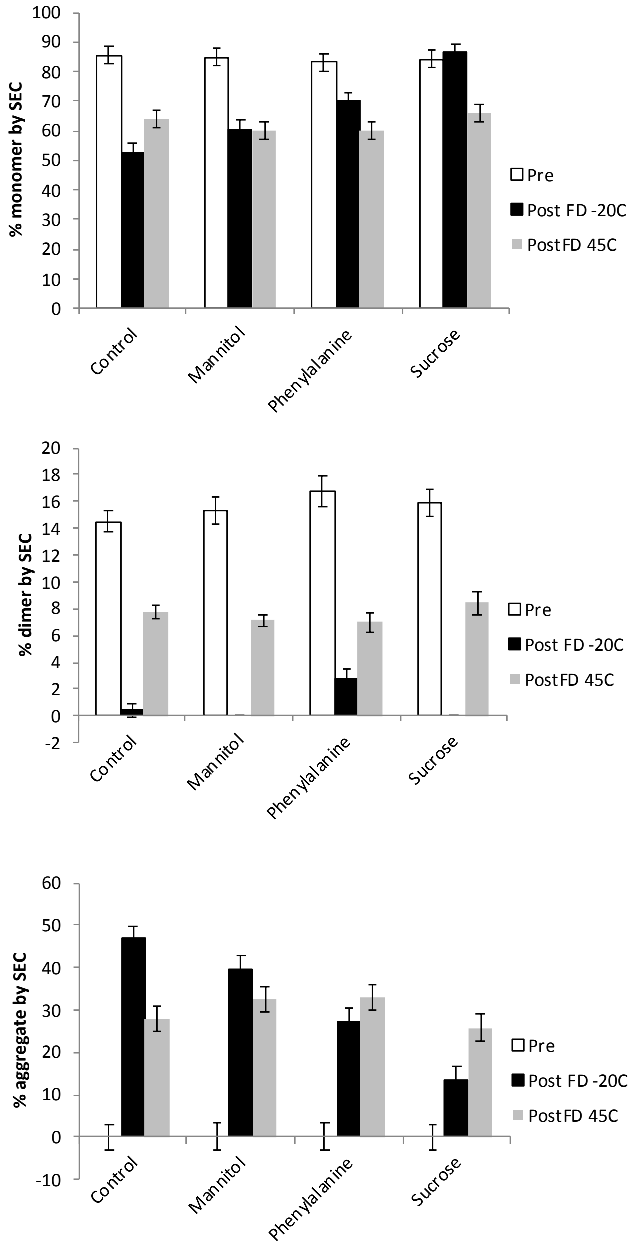

Figure S4. Impact of excipients on monomer, dimer and aggregate content for G-CSF for mannitol, phenylalanine and sucrose pre- and post- lyophilisation. A) Monomer content determined by SEC before (white) and after lyophilisation and storage at -20 °C (black) or at 45 °C (light grey) for 30 days. Samples contained 0.3 mg/ml G-CSF in 50 mM citric acid pH 4.25, with no excipient (control), or either 1% (w/v) mannitol, phenylalanine or sucrose. Measurements made from three independently processed samples and error bars denote standard deviations. Aggregate content was determined as 100%-%monomer-%dimer as aggregates did not result in peaks on the SEC column due to removal either by pre-centrifugation or due to trapping on the column.
